# Supplementary material for: Subject specific muscle synergies and mechanical output during cycling with arms or legs
Source: PeerJ. 2022 Mar 29;10:e13155. doi: 10.7717/peerj.13155 (PMC8973464; doi:10.7717/peerj.13155)
Supplement: Supplemental Information 1 [file peerj-10-13155-s001.pdf]

Data for upper limbs (U) or lower limbs (L) cycling for each subject :
- 1 Lode.csv for torque data
- 1 c3d for kinematics data
- 2 csv for EMG data (Wingate + experiment)

Codebook:
In Lode.csv :
- Complexe = revolution
- Heure de début = Starting timing
- Puissance moyenne = Mean power
- TPM moyen = mean rotation per minute
- Couple de pédale gauche/droit = left/right crank torque
- Heure du delta = timing apparition of the sample during the revolution

In emg.csv :
The first 4 columns concern the Quattro electrodes.
For Legs, corresponding muscles are : SOL / GL / GM / TA
For arms, corresponding muscles are : FDS / FCR / BR / ECRL
