# Supplementary material for: Subject specific muscle synergies and mechanical output during cycling with arms or legs
Source: PeerJ. 2022 Mar 29;10:e13155. doi: 10.7717/peerj.13155 (PMC8973464; doi:10.7717/peerj.13155)

UPPER LIMBS

Pairwise correlations of each variable.

Bottom corner: graphical representation. Each point represents the occurrence of a cycle for each participant. A linear regression is shown. Upper corner: associated r, r2 and p-value. Diagonal: distribution of each variable.

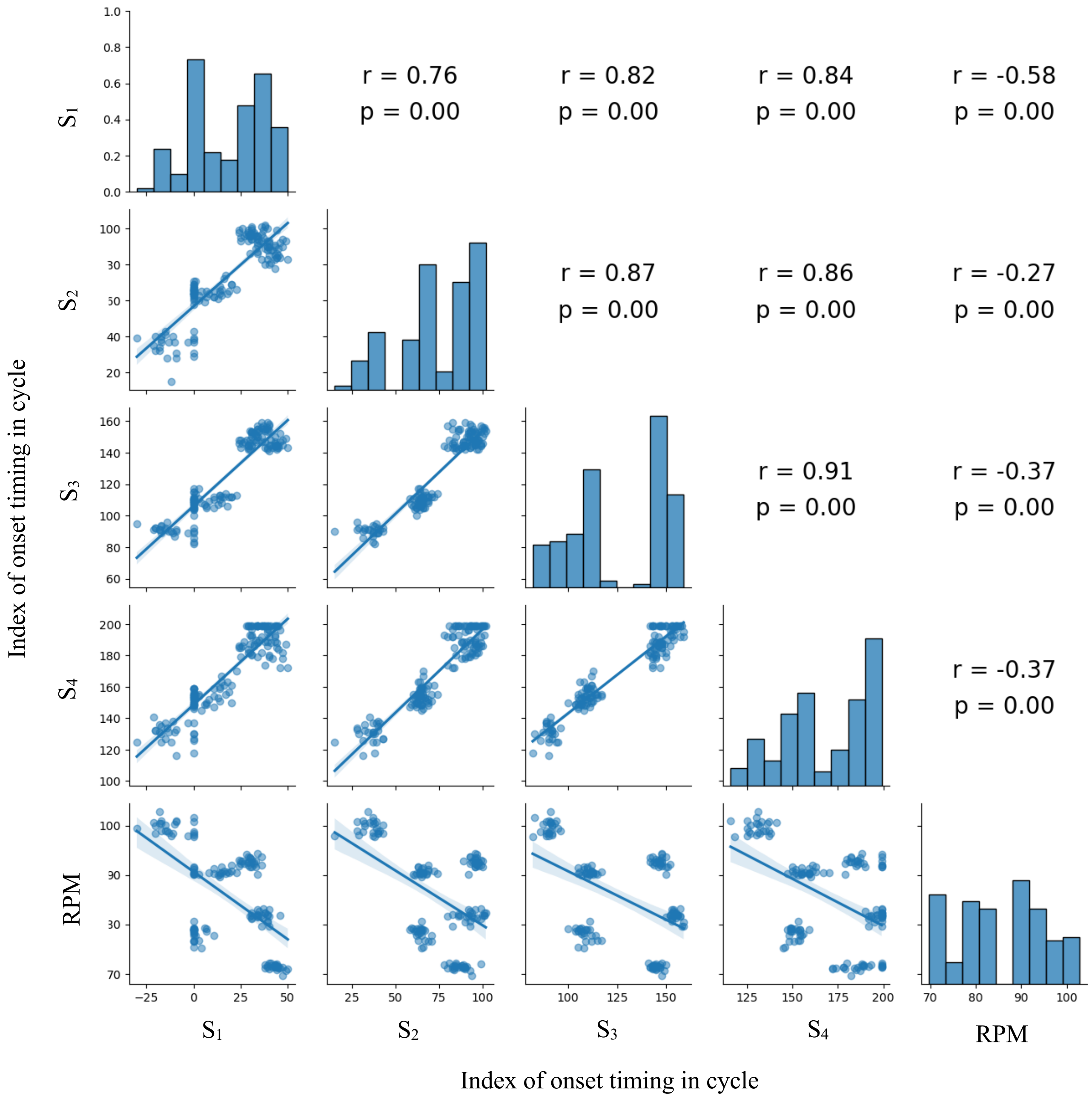

Supplement: Supplemental Information 2 — Bottom corner: graphical representation. Each point represents the occurrence of a cycle for each participant. A linear regression is shown. Upper corner: associated r, r2 and p-value. Diagonal: distribution of each variable. S1-4: synergies 1 to 4; RPM: cadence. [file peerj-10-13155-s002.pdf]
