# Supplementary figures and images for: Subject specific muscle synergies and mechanical output during cycling with arms or legs
Source: PeerJ. 2022 Mar 29;10:e13155. doi: 10.7717/peerj.13155 (PMC8973464; doi:10.7717/peerj.13155)

Normalized EMG

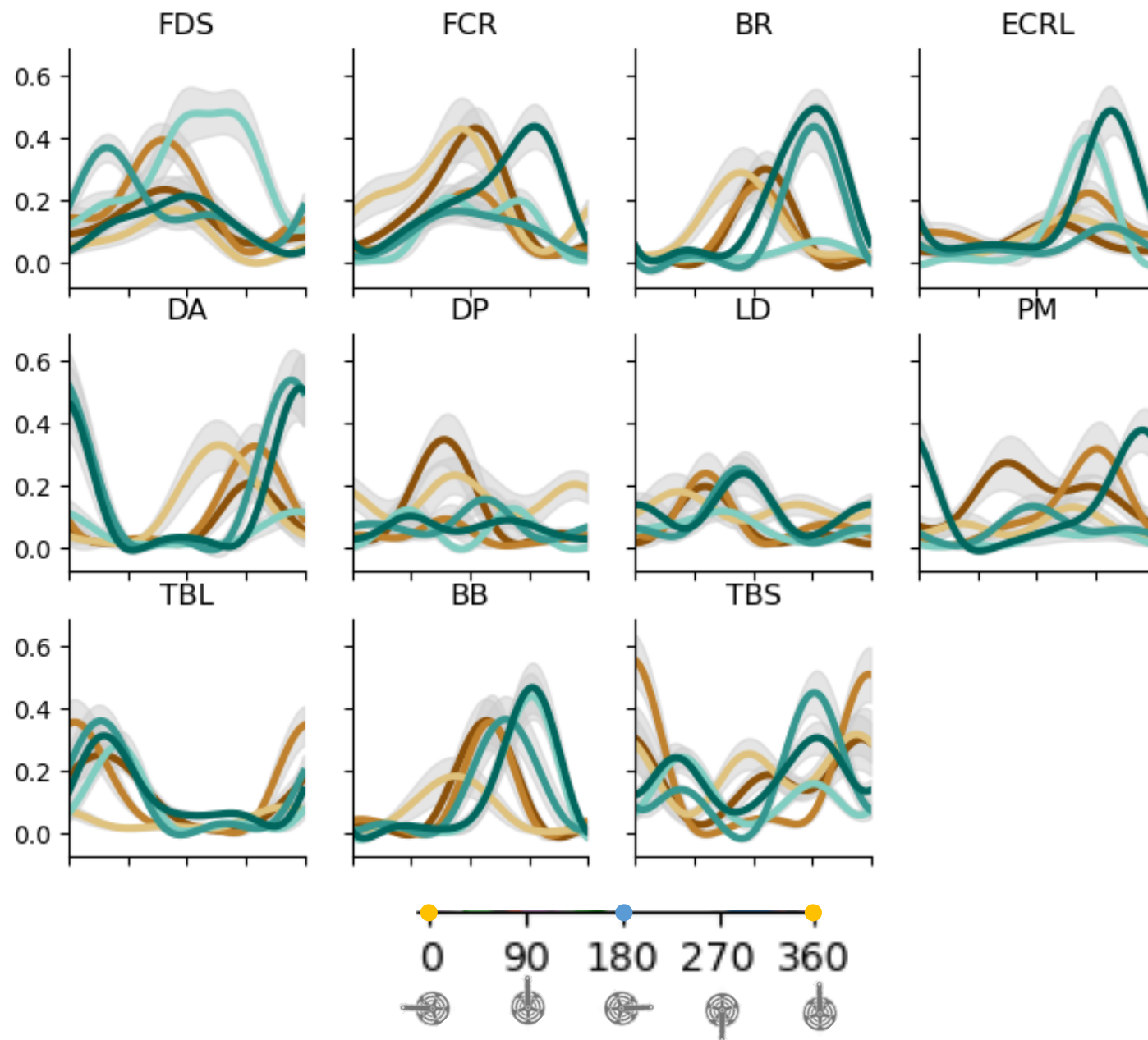

Supplement: Supplemental Information 4 — Mean ±sd patterns of EMG for each participant across de cycling revolution. UL : upper limbs [file peerj-10-13155-s004.pdf]

Normalized EMG

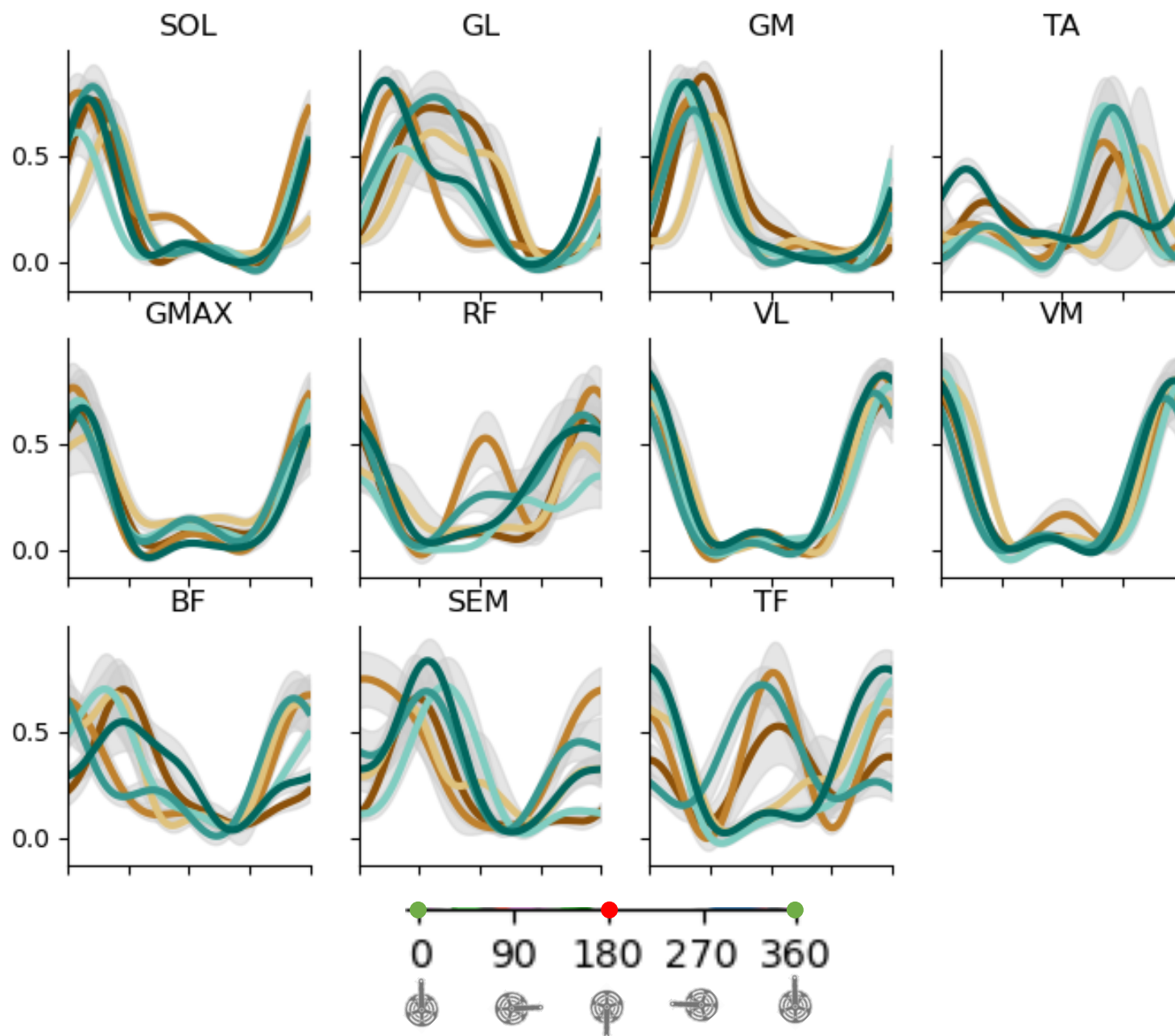

Supplement: Supplemental Information 5 — Mean ±sd patterns of EMG for each participant across de cycling revolution. LL : Lower limbs. [file peerj-10-13155-s005.pdf]
